# Different clinical courses of central precocious pubertal girls according to the age at presentation and treatment

**DOI:** 10.1186/1687-9856-2013-S1-P69

**Published:** 2013-10-03

**Authors:** Heon-Seok Han, Jae Hong Yu

**Affiliations:** 1 Department of Pediatrics, Chungbuk National University Hospital, Cheongju, Korea; 2 Joy Children's Hospital, Daejeon, Korea

## 

The progressivity of central precocious puberty (CPP) seemed to depend on the age at presentation. We investigated the clinical courses between the early onset and late onset groups.

One hundred thirty five girls with central precocious puberty diagnosed between Jan. 2003 and Dec. 2009 were included. Among 135 patients, 123 were idiopathic CPP (91.1%) and twelve (8.89%) had neurogenic problems, such as arachnoid cyst, hydrocephalus, pineal cyst, pituitary cyst, partial empty sella, cerebral palsy, diffuse cortical atrophy, tuberous sclerosis, and Langerhans cell histiocytosis. Also noted was small for gestational age in another 12 patients among the idiopathic patients. CPP criteria were development of secondary sexual characteristics before 8 yr of chronologic age (CA) with advanced bone age (BA), and stimulated LH > 5 IU/L. They were treated with GnRH analogues every 4 weeks and followed for more than 1 yr. According to the age at initiation of treatment, 9 were before 6 yr, 11 were 6~7 yr, 61 were 7~8 yr, 54 were > 8 yr. Subjects were divided into two groups if they were treated before (Group I) or after 7 yr of age (Group II). Clinical courses and laboratory findings were evaluated every 6 months. We compared anthropometric parameters, calculated predicted adult height (PAH), predicted treatment periods, and laboratory findings between the two groups.

Among the baseline parameters, BA and CA were greater in group II, but BA/CA ratio were significantly greater in group I. The time needed for disappearing the CA and BA difference was 4.64 yr (3.74~5.54 yr) for the total patients, but the time is significantly different between the two groups, 7.98 yr (3.88~12.07 yr) for group I and 4.24 yr (3.74~4.73 yr) for group II. The time needed for disappearing the PAH and TH difference was 2.49 yr (1.96~3.01 yr), but the time is significantly different between the groups, 4.37 yr (0~12.58 yr) for group I and 2.32 yr (1.48~3.16 yr) for group II.

Among the girls with CPP, younger age group had more advanced bone age compared to chronologic age, and needed significantly longer treatment periods for the disappearance of BA-CA gap and PAH-TH gap.

